# Functional diversity of staphylococcal surface proteins at the host-microbe interface

**DOI:** 10.3389/fmicb.2023.1196957

**Published:** 2023-05-18

**Authors:** Nicoletta Schwermann, Volker Winstel

**Affiliations:** ^1^Research Group Pathogenesis of Bacterial Infections, TWINCORE, Centre for Experimental and Clinical Infection Research, a Joint Venture Between the Hannover Medical School and the Helmholtz Centre for Infection Research, Hannover, Germany; ^2^Institute of Medical Microbiology and Hospital Epidemiology, Hannover Medical School, Hannover, Germany

**Keywords:** *Staphylococcus aureus*, surface proteins, sortase A, abscess, vaccine

## Abstract

Surface proteins of Gram-positive pathogens are key determinants of virulence that substantially shape host-microbe interactions. Specifically, these proteins mediate host invasion and pathogen transmission, drive the acquisition of heme-iron from hemoproteins, and subvert innate and adaptive immune cell responses to push bacterial survival and pathogenesis in a hostile environment. Herein, we briefly review and highlight the multi-facetted roles of cell wall-anchored proteins of multidrug-resistant *Staphylococcus aureus*, a common etiological agent of purulent skin and soft tissue infections as well as severe systemic diseases in humans. In particular, we focus on the functional diversity of staphylococcal surface proteins and discuss their impact on the variety of clinical manifestations of *S. aureus* infections. We also describe mechanistic and underlying principles of staphylococcal surface protein-mediated immune evasion and coupled strategies *S. aureus* utilizes to paralyze patrolling neutrophils, macrophages, and other immune cells. Ultimately, we provide a systematic overview of novel therapeutic concepts and anti-infective strategies that aim at neutralizing *S. aureus* surface proteins or sortases, the molecular catalysts of protein anchoring in Gram-positive bacteria.

## Introduction

*Staphylococcus aureus* is a notorious pathogen that causes fatal diseases in the human population (Lowy, 1998; Lee et al., 2018). This microbe is a leading causative agent of skin and soft tissue infections (SSTIs), pneumonia, endocarditis, septic arthritis, osteomyelitis, bacteremia, and sepsis (Lowy, 1998; Kuehnert et al., 2006; Klevens et al., 2007). Of note, a global survey indicates that this pathogen accounts for more than 1 million deaths annually (GBD 2019 Antimicrobial Resistance Collaborators, 2022), an alarming death count which undoubtedly correlates with multidrug resistance (Chambers and Deleo, 2009; Lee et al., 2018), genetic flexibility and adaptive evolution (Chambers and Deleo, 2009; Malachowa and DeLeo, 2010; Smith et al., 2022; Howden et al., 2023), as well as refined immuno-evasive maneuvers this microbe evolved to overcome host immunity (Spaan et al., 2013; Thammavongsa et al., 2015a). Specifically, *S. aureus* secretes an extraordinary repertoire of virulence factors into the environment in order to establish acute and persistent infections in mammalian hosts (Foster, 2005; Thammavongsa et al., 2015a). Examples involve pore-forming and cytolytic toxins, superantigens, and multiple immuno-modulatory exoenzymes, which harbor an N-terminal signal peptide required for a Sec-machinery-dependent translocation across the cytoplasmic membrane (Foster, 2005; Spaan et al., 2013; Thammavongsa et al., 2015a; Tam and Torres, 2019). Moreover, *S. aureus* expresses up to 24 signal peptide-bearing and pathogenicity-associated cell surface proteins that are characterized by diverse functional domains and flexible host ligand binding properties, as well as by a short C-terminal sorting sequence (Foster, 2019; Schneewind and Missiakas, 2019). This sequence, typically an LPXTG motif (Schneewind et al., 1992; Schneewind and Missiakas, 2019), is sensed and cleaved by sortase A (SrtA), a type II membrane protein and transpeptidase that catalyzes anchoring of cell surface proteins to the peptidoglycan of *S. aureus* and other Gram-positive bacteria (Mazmanian et al., 1999; Ton-That et al., 1999; Schneewind and Missiakas, 2019). Remarkably, *S. aureus srtA* mutants largely fail to colonize the host and are strongly attenuated in animal models of infectious disease (Mazmanian et al., 2000; Jonsson et al., 2003; Schaffer et al., 2006; Bubeck Wardenburg et al., 2007; Weidenmaier et al., 2008; Cheng et al., 2009; Chen et al., 2014; Misawa et al., 2015), a striking phenotype that inspired the staphylococcal research community to examine the individual roles of cell surface-displayed proteins at the host-microbe interface.

Herein, we briefly summarize the multi-facetted and sometimes redundant functions of cell surface proteins during local and invasive *S. aureus* infections. We also discuss how these proteins affect staphylococcal immune evasion and interaction with professional and non-professional phagocytes. Ultimately, we highlight the potential role of staphylococcal surface proteins in the design of vaccines, unique anti-infective agents, and novel therapeutic intervention strategies.

## Role of staphylococcal surface proteins during colonization and establishment of skin and soft tissue infections

**Figure 1:**
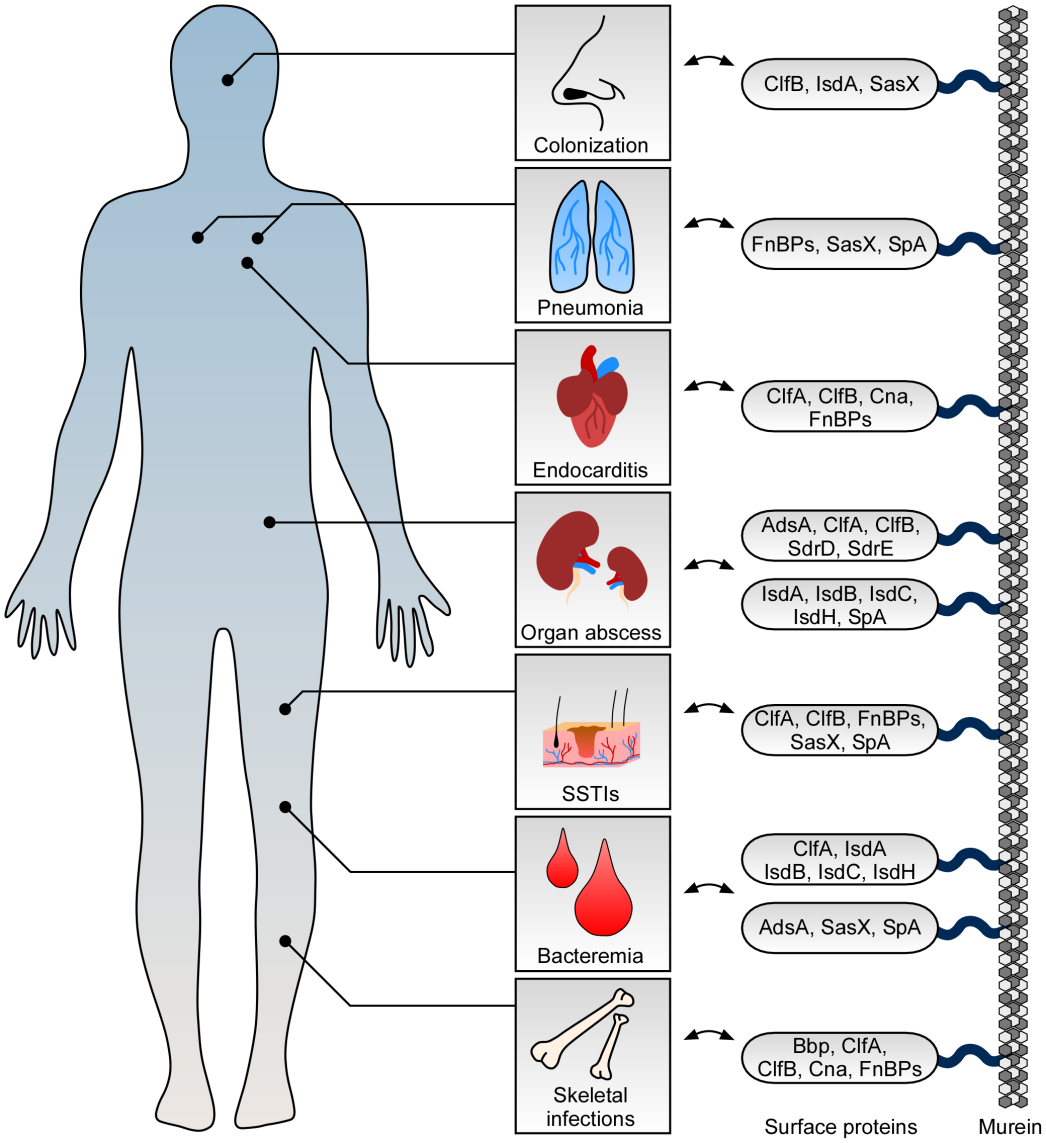
Role of staphylococcal surface proteins at the host-microbe interface. *Staphylococcus aureus* deploys surface proteins to promote interaction with mammalian hosts. While some surface proteins affect *S. aureus* nasal colonization, others contribute to skin and soft tissue infections (SSTIs) and fatal invasive diseases. Key surface proteins including adenosine synthase A (AdsA), bone sialoprotein (Bbp), clumping factor A and B (ClfA and ClfB), collagen adhesin (Cna), fibronectin-binding proteins (FnBPs), iron-regulated surface determinant proteins (IsdABCH), *S. aureus* surface protein X (SasX), serine aspartate repeat containing proteins D and E (SdrD and SdrE), and staphylococcal protein A (SpA) along with their proposed function during colonization and infection of human hosts are highlighted.

*S. aureus* is a very frequent cause of SSTIs which include cellulitis, inflamed hair follicles (folliculitis), furuncles and deep-seated abscesses, and surgical site infections (Lowy, 1998; Lee et al., 2018). During the establishment of these infections, cell surface proteins play a substantial role and largely contribute to initial adhesion and invasion of host cells (Foster et al., 2014; Lee et al., 2018; Foster, 2019). For example, several staphylococcal surface proteins including clumping factor B (ClfB), fibronectin-binding protein B (FnBPB), and iron-regulated surface determinant protein A (IsdA) mediate binding to human loricrin (Clarke et al., 2009; Mulcahy et al., 2012; da Costa et al., 2022), the most abundant protein of the cornified cell envelope and terminally differentiated corneocytes (Candi et al., 2005). Thus, it is not unexpected that some surface proteins influence staphylococcal colonization of the nasal cavity which is the natural niche of *S. aureus* (Figure 1; O’Brien et al., 2002; Clarke et al., 2006; Wertheim et al., 2008; Mulcahy et al., 2012; Weidenmaier et al., 2012; Sun et al., 2018). Specifically, ClfB- and IsdA-mediated binding to loricrin has been shown to affect interaction with squamous nasal epithelial cells thereby facilitating stable colonization of rodent or human nares (Clarke et al., 2006, 2009; Wertheim et al., 2008; Mulcahy et al., 2012). This process is further strengthened by IsdA-mediated interaction with involucrin and cytokeratin-10 as well as other staphylococcal surface proteins such as serine aspartate repeat containing protein D (SdrD) and *S. aureus* surface protein G (SasG) which also confer attachment to desquamated epithelial cells (Clarke et al., 2009; Corrigan et al., 2009; Askarian et al., 2016; Mills et al., 2022). Nonetheless, colonization and initial binding to upper skin layers not necessarily correlate with establishment of purulent infections of the skin. Albeit colonization of the host is generally accepted to be a risk factor for acquiring local and invasive staphylococcal diseases (von Eiff et al., 2001; Wertheim et al., 2005), establishment of these infections often requires skin lesions, wounds, or other medical conditions that favor pathogen entry (Cheng et al., 2011; Tong et al., 2015). For example, patients with atopic dermatitis, a chronic inflammatory skin disease associated with an IgE-mediated allergic response (Bieber, 2008; Werfel, 2009), are at elevated risk to be colonized with *S. aureus* and therefore often suffer from local infections of the skin (Geoghegan et al., 2018; Ogonowska et al., 2020). During atopic dermatitis, ClfB and particularly fibronectin-binding proteins (FnBPs) not only mediate binding to skin cells but also react with IgE antibodies thereby triggering specific inflammatory and allergic immune responses (Cho et al., 2001; Reginald et al., 2011; Fleury et al., 2017; Farag et al., 2022). In that regard, we further note that ClfB contributes to SSTIs and early stages of abscess formation in experimental skin infection models (Figure 1; Lacey et al., 2019). Mice subcutaneously infected with a *clfB* mutant of the *S. aureus* MRSA isolate USA300 developed smaller skin lesions over the course of the infection as compared to animals infected with the parental strain (Lacey et al., 2019). This phenomenon is associated with loricrin, which was found to be a component of the abscess wall and major host factor required for the development of skin lesions in mammals (Lacey et al., 2019). Likewise, bacterial mutants lacking FnBPs exhibited attenuated virulence in skin abscess models (Kwiecinski et al., 2014), probably also as a result of impaired host cell invasion and altered interaction with loricrin or extracellular matrix components (Fowler et al., 2000; Edwards et al., 2011; da Costa et al., 2022). Reduced bacterial loads in these models may further be explained by FnBPB-mediated neutralization of histones (Pietrocola et al., 2019), an antimicrobial component of neutrophil extracellular traps (NETs) which are formed in response to *S. aureus* during infection of the skin or other body parts (Brinkmann et al., 2004; Yipp et al., 2012; von Kockritz-Blickwede and Winstel, 2022). With this in mind, it is also worth noting that various other *S. aureus*-derived surface proteins assist in protecting staphylococci against professional phagocytes thereby essentially contributing to the development of abscesses and SSTIs (Foster, 2019; Schneewind and Missiakas, 2019). In particular, staphylococcal protein A (SpA) is a chief factor required for proper abscess formation in the skin as staphylococcal mutants lacking this determinant display virulence defects and reduced abscess volume in experimental murine models of skin infection (Patel et al., 1987; Kwiecinski et al., 2014). Moreover, clumping factor A (ClfA) has been linked to skin infections inasmuch as subcutaneous abscesses from rabbits infected with *clfA*-deficient staphylococci differed in size and had only weak evidence of vasculitis and thrombosis when compared to lesions formed by the parental *S. aureus* isolate (Malachowa et al., 2016). Thus, SpA and ClfA influence the pathogenesis of skin abscesses and associated SSTIs, presumably due to their anti-phagocytic properties which are known to promote staphylococcal evasion from neutrophil-mediated killing (Dossett et al., 1969; Higgins et al., 2006). However, protein A was also found to affect infections of the skin by modulating inflammatory signaling cascades and cell death modalities in neutrophils and epithelial cells, highlighting the multi-facetted functions of staphylococcal cell surface proteins during establishment of SSTIs (Classen et al., 2011; Soong et al., 2012; Gonzalez et al., 2019; Ledo et al., 2020). Lastly, we note that not all cell surface proteins impacting SSTIs are part of the staphylococcal core genome. Specific MRSA clones with the sequence type ST239, for instance, carry a large ΦSPβ-like prophage in their genome that encodes a unique cell wall-anchored protein termed SasX (Li et al., 2012). Of note, mutant bacteria lacking *sasX* failed to colonize the nares of mice and were attenuated during experimental skin infection, a pioneering observation that has been linked to MRSA spread in China and other Asian countries (Li et al., 2012; Liu et al., 2015). Together, this comprehensive work underscores the relevance and importance of cell surface-displayed proteins during *S. aureus* colonization of host tissues and infections of the skin.

## Impact of cell surface proteins on *Staphylococcus aureus* bacteremia and intra-organ abscess formation

*S. aureus* is also a frequently encountered agent of invasive and life-threatening diseases (Lowy, 1998; Lee et al., 2018). Bacteremia, for example, is a serious medical condition associated with high morbidity and mortality rates that often occurs upon staphylococcal entry into the bloodstream (Thomer et al., 2016; Bai et al., 2022). But how does *S. aureus* manage to survive within this hostile environment? Earlier studies and particularly transcriptional profiling of *S. aureus* in human blood identified multiple staphylococcal virulence determinants that became highly expressed under bacteremia-mimicking conditions (Malachowa et al., 2011). Among these factors are secreted toxins and various cell surface proteins including IsdA, IsdB, and IsdC, all elements of the iron-regulated surface determinant system (Isd; Figure 1; Malachowa et al., 2011). This system is required for iron and heme uptake in staphylococci and thus helps *S. aureus* to overcome iron limitation in the host (Mazmanian et al., 2003; Hammer and Skaar, 2011). Accordingly, *S. aureus* mutants lacking IsdA, IsdB, IsdC, as well as IsdH exhibit decreased survival in blood and virulence defects in mouse models of bloodstream infection (Cheng et al., 2009; Visai et al., 2009; Kim et al., 2010b). This also holds true for staphylococcal variants that fail to express protein A, SasX, or adenosine synthase A (AdsA; Palmqvist et al., 2002; Thammavongsa et al., 2009; Li et al., 2012; Falugi et al., 2013). While protein A and SasX predominantly aid in preventing phagocytic clearance of *S. aureus* by either capturing immunoglobulins or promoting intercellular aggregation (Forsgren and Sjoquist, 1966; Dossett et al., 1969; Forsgren and Quie, 1974; Li et al., 2012), AdsA along with its 5′–3′-nucleotidase activity dampens neutrophil responses and coupled killing of *S. aureus* during acute bloodstream infection by converting host-derived adenosine monophosphate into immuno-suppressive adenosine (Thammavongsa et al., 2009). Nevertheless, entry and survival of *S. aureus* in blood causes organism-wide dissemination and formation of new replicative niches that often manifest as abscesses (Thomer et al., 2016). Establishment of these lesions can occur in almost all organs and requires, *inter alia*, the activity of specific cell surface proteins (Cheng et al., 2011; Thomer et al., 2016). For example, elements of the Isd machinery, ClfA and ClfB, as well as protein A significantly contribute to intra-organ abscess formation and priming of persistent infections (Cheng et al., 2009). Moreover, lack of SdrD, a cell wall-anchored protein that is only prevalent in approximately 60% of all *S. aureus* isolates (Sabat et al., 2006), dramatically lowered abscess formation and bacterial loads in organ tissues in murine models of systemic infection (Cheng et al., 2009; Askarian et al., 2017). Likewise, genetic ablation of *adsA* decreased the staphylococcal burden in renal tissues following intravenous challenge and concurrently ameliorated overall disease outcomes in mice (Thammavongsa et al., 2009). However, this phenomenon not only correlated with the failure of *adsA*-deficient staphylococci to synthesize adenosine during the initial phase of a bloodstream infection. Previous work showed that AdsA, together with the help of a secreted nuclease, converts NETs and host-derived DNA molecules into phagocyte-eliminating deoxyadenosine and deoxyguanosine, two purine effector-deoxyribonucleosides that promote killing of immune cells by targeting the purine salvage pathway-apoptosis axis (Thammavongsa et al., 2013; Winstel et al., 2018, 2019; Tantawy et al., 2022). Following this strategy, phagocyte entry into purulent cavities of deep-seated abscesses is efficiently suppressed thereby enhancing staphylococcal survival and establishment of persistent infections in organ tissues (Thammavongsa et al., 2013; Winstel et al., 2019). Thus, staphylococcal surface proteins essentially contribute to *S. aureus* bloodstream infection and intra-organ abscess development.

## Contribution of staphylococcal surface proteins to skeletal infections, endocarditis, and pneumonia

Not all of the aforementioned cell surface proteins exclusively affect abscess formation upon bloodstream infection and staphylococcal dissemination in the host (Figure 1; Foster, 2019). ClfA and protein A, for instance, play a key role during septic arthritis (Josefsson et al., 2001; Palmqvist et al., 2002), a dangerous joint disease which is characterized by fever, joint pain and swelling, as well as redness of affected body regions (Shirtliff and Mader, 2002; Mathews et al., 2010). Development of septic arthritis is also linked to the expression of staphylococcal collagen adhesin (Cna), a protein and member of the MSCRAMM (microbial surface component recognizing adhesive matrix molecule) family that mediates binding to collagen and cartilage (Patti et al., 1994; Xu et al., 2004). Moreover, fibrinogen-and fibronectin-binding proteins (i.e., ClfA, ClfB, FnBPA, and FnBPB) promote bacterial aggregation in human synovial fluid, a biofilm-like state that protects *S. aureus* from antibiotics and phagocytes within the joint cavity (Dastgheyb et al., 2015). In this regard, we further note that some of these proteins impact staphylococcal skeletal infections and chronic bone diseases (i.e., osteomyelitis; Gimza and Cassat, 2021; Masters et al., 2022). For example, *S. aureus* Cna and bone sialoprotein (Bbp), another MSCRAMM that facilitates adhesion to fibrinogen (Vazquez et al., 2011), confer binding to the bone matrix and thus contribute to the pathogenesis of osteomyelitis (Ryden et al., 1989; Elasri et al., 2002; Campoccia et al., 2009; Persson et al., 2009). Likewise, protein A is a major modulator of this disease as binding of SpA to osteoblasts prevents cellular proliferation and stimulates apoptotic cell death in bone-synthesizing cells (Claro et al., 2011; Widaa et al., 2012). Development of osteomyelitis and establishment of replicative niches in the bone environment is further promoted by FnBPs (Ahmed et al., 2001), crucial *S. aureus* surface proteins that also impact non-osseous and fatal staphylococcal diseases of the heart (Foster, 2019). More specifically, FnBPs along with fibrinogen-and collagen-binding proteins of *S. aureus* influence the pathogenesis of infective endocarditis (Kuypers and Proctor, 1989; Moreillon et al., 1995; Hienz et al., 1996; Entenza et al., 2000; Que et al., 2005; Claes et al., 2017), a serious and life-threatening disease affecting the endocardial surface of the heart (Holland et al., 2016). Mechanistically, these proteins promote attachment of *S. aureus* to vessel walls, thrombi, and traumatized or inflamed heart tissues (Kuypers and Proctor, 1989; Moreillon et al., 1995; Entenza et al., 2000; Que et al., 2005; Claes et al., 2017). At later stages, FnBPA together with other virulence factors trigger staphylococcal invasion of the valve endothelium thereby aiding in the establishment of novel proliferative sites that provoke tissue destruction, cardiac abscess formation, and organ failure (Hamill et al., 1986; Que et al., 2005; Holland et al., 2016). Not surprisingly perhaps that FnBPs have a similar role during acute lower respiratory tract infection (pneumonia) as these factors confer binding to and uptake of *S. aureus* into airway epithelial cells (Figure 1; McElroy et al., 2002; Mongodin et al., 2002). Yet, failure to enter host cells due to missing expression of FnBPs may also boost staphylococcal pathogenicity as demonstrated in a rat model of experimental pneumonia (McElroy et al., 2002). Presumably, intracellular replication and persistence is favored by specific *S. aureus* isolates and might help to better adapt to the inflamed lung environment. This is also exemplified by the persistent lifestyle of staphylococcal small colony variants (SCVs), an auxotrophic and hard-to-treat subpopulation of *S. aureus* that often emerges during airway infections and in patients with cystic fibrosis (Proctor et al., 2006; Kahl et al., 2016). SCVs particularly aim at infiltrating host cells by upregulating FnBPs and other cell surface proteins to establish a protective niche that shields the microbe from neutrophils and alveolar macrophages (Vaudaux et al., 2002; Kahl et al., 2005; Mitchell et al., 2008; Tuchscherr et al., 2010, 2011; Kriegeskorte et al., 2014). Since SCVs as well as wildtype *S. aureus* often co-infect the lung together with other pathogens (McCullers, 2014; Oliva and Terrier, 2021), we finally appreciate that staphylococcal surface proteins may even impact outcomes of polymicrobial infections. Most notably, recent advances suggest that *S. aureus* IsdA manipulates the Janus kinase-signal transducer and activator of transcription (JAK–STAT) signaling cascade thereby accelerating proliferation of severe acute respiratory syndrome coronavirus type 2 (SARS-CoV-2) in epithelial cells (Goncheva et al., 2023). Moreover, protein A was found to protect *Pseudomonas aeruginosa* from neutrophil-mediated killing and altered the capacity of this microbe to form biofilms (Armbruster et al., 2016). This mechanism involves binding of protein A to cell surface structures of *P. aeruginosa* and the release of SpA from the staphylococcal cell wall, an earlier described phenomenon that may even be linked to binding of protein A to tumor necrosis factor receptor 1 (TNFR1) on lung epithelial cells thereby shaping staphylococcal pneumonia (Gomez et al., 2004; Becker et al., 2014; Armbruster et al., 2016). Overall, these compelling studies highlight the variable functions of cell surface proteins during *S. aureus* bone and joint infections, endocarditis, and pneumonia.

## Targeting cell surface proteins and sortase A to improve *Staphylococcus aureus* infection outcomes

Due to their near-essential role during *S. aureus* pathogenesis and colonization of the host, cell surface proteins represent attractive targets for the development of new prophylactic and anti-infective intervention strategies. Earlier studies demonstrated that vaccination of laboratory animals with staphylococcal cell surface proteins together with passive immunization approaches confer protective effects against *S. aureus* disease (Table 1). For example, IsdA-or IsdB-based immunization of mice and interference with heme-iron uptake attenuated the adaptive properties and virulence potential of staphylococci in multiple *in vivo* models (Table 1; Clarke et al., 2006; Kuklin et al., 2006; Brown et al., 2009; Kim et al., 2010b; Bennett et al., 2019a,b). Likewise, immunization of mice with SpA_KKAA_, a non-toxigenic protein A-based vaccine (Kim et al., 2010a), or safety-improved variants thereof abolished staphylococcal pathogenicity in murine and guinea pig models of bloodstream infection, and even promoted decolonization of rodent nares (Table 1; Kim et al., 2010a, 2015; Sun et al., 2018; Shi et al., 2021). Moreover, SpA-targeting monoclonal antibodies (mAbs) and derived humanized variants displayed therapeutic activity in abscess mouse models and concurrently offered protection against bacteremia and neonatal sepsis (Table 1; Kim et al., 2012; Thammavongsa et al., 2015b; Chen et al., 2019, 2020, 2022). These effects correlated with the antibody-mediated neutralization of the immunoglobulin Fcγ-binding and B-cell receptor crosslinking properties of SpA and enhanced opsonophagocytic killing of staphylococci in mouse or human blood (Kim et al., 2012; Thammavongsa et al., 2015b; Chen et al., 2019, 2020, 2022). Accelerated killing of *S. aureus* in host blood paired with ameliorated outcomes of septic arthritis or bacteremia was also observed in passively immunized animals that received ClfA-or SraP (serine-rich adhesin for platelets)-targeting antibodies (Josefsson et al., 2001; Tkaczyk et al., 2016; Yang et al., 2016; Zhou et al., 2021). Administration of an antiserum raised against staphylococcal AdsA further aided in rescuing mice from fatal bloodstream infection and peritonitis, presumably as a result of enhanced killing of staphylococci by circulating neutrophils that can no longer be suppressed by pathogen-derived adenosine (Zhang et al., 2017b). Accordingly, cell surface proteins and their immunogenic potential have often been exploited to formulate an effective vaccine against *S. aureus* (Clegg et al., 2021). Examples involve a recombinant, protein A-and IsdB-N2-containing five-antigen *S. aureus* vaccine (rFSAV) as well as SA4Ag, a multicomponent vaccine composed of capsular polysaccharide conjugates and recombinant forms of ClfA and the staphylococcal manganese transporter C (MntC; Creech et al., 2017; Frenck et al., 2017; Zeng et al., 2020). However, various clinical trials ended in failure due to adverse effects or limited efficacy in diseased patients (Clegg et al., 2021), thereby asking for improved vaccination strategies that may encompass probiotic-based immunization (Pan et al., 2023), advanced antibody engineering (Chen et al., 2020, 2022), or usage of live-attenuated vaccine platforms (Cabral et al., 2017; Moscoso et al., 2018). Alternatively, chemical interference with the transpeptidase activity of sortase A may also help to limit *S. aureus* colonization and severity of staphylococcal disease. In fact, previous work demonstrated that small molecule inhibitor-based blockade of sortase A can reduce *S. aureus* virulence in different animal model of infectious disease (Table 1). Computational drug engineering, for instance, identified 3,6-disubstituted triazolothiadiazole as a potent inhibitor of sortase A that improved infection outcomes of lethal *S. aureus* bacteremia (Table 1; Zhang et al., 2014). Further, compound library screening helped to isolate several natural products with sortase A-blocking and anti-infective properties (Table 1; Song et al., 2022a,b). Some of these agents even potentiated the efficacy of cell wall biosynthesis-targeting antibiotics during experimental pneumonia, presumably aiding in the design of poly-therapeutic approaches that may also encompass usage of allantodapsone, a prototype pan-inhibitor of staphylococcal adhesion to extracellular matrix proteins (Prencipe et al., 2022), to combat complicated MRSA infections in the future (Table 1; Song et al., 2022a,b; Wang et al., 2022b).

**Table 1:** Selected mono-therapeutic approaches to attenuate *Staphylococcus aureus* pathogenicity *in vivo.*

Target	Therapeutic approach^a^^,^ ^b^^,^ ^c^^,^ ^d^	Effect^e^	References
AdsA	Vaccination with rAdsA or α-AdsA rabbit serum	therapeutic effect in peritonitis, survival, and skin abscess mouse models	Zhang et al. (2017b)
ClfA	Immunization of laboratory animals with rClfA or α-ClfA antibodies	reduces severity of *S. aureus*-mediated septic arthritis; protective effect in bacteremia and prosthetic-device infection models	Josefsson et al. (2001), Arrecubieta et al. (2008), Tkaczyk et al. (2016)
	Application of humanized mAb targeting ClfA	offers protection in a rabbit model of infective endocarditis	Domanski et al. (2005)
	Vaccine approach by using a ClfA-specific murine mAb	attenuates *S. aureus* virulence in a mouse sepsis model	Hall et al. (2003)
ClfB	Vaccination with UV-killed *S. aureus*, rClfB, or a ClfB-targeting antibody	abolishes nasal colonization in mice; protects against *S. aureus* skin infection	Schaffer et al. (2006), Lacey et al. (2019)
Cna	Immunization with rCna or α-Cna rat serum	protects from *S. aureus* infection and reduces mortality of mice upon lethal challenge	Nilsson et al. (1998)
FnBPA	Administration of FnBPA fusion proteins or rFnBPA for vaccination purposes	ameliorates outcomes of experimental mastitis in mice; protective effect in lethal challenge mouse model; reduced bacterial loads in organ tissues	Mamo et al. (1994), Zuo et al. (2014)
IsdA	Application of human mAb specific for IsdA	decreases bacterial loads in a murine model of systemic infection	Bennett et al. (2019a)
	Exploitation of purified and IsdA-specific rabbit antibody	lowers bacterial loads in a renal abscess mouse model; protective effect upon lethal *S. aureus* infection	Kim et al. (2010b)
	Vaccine approach with purified IsdA	diminishes nasal colonization of cotton rat nares	Clarke et al. (2006)
IsdB	Vaccination of mice with rIsdB	improves survival of mice upon lethal challenge with *S. aureus*	Kuklin et al. (2006)
	Application of probiotic-based vaccine (WXD171-IsdB)	mediates protection from *S. aureus* in skin, pneumonia, and sepsis mouse models	Pan et al. (2023)
	Immunization of mice with human mAb binding to IsdB-NEAT2	attenuates *S. aureus* virulence in a murine septic model	Bennett et al. (2019b)
	Purified rabbit antibody specific for IsdB	decreases bacterial loads in a renal abscess mouse model; protects mice from lethal *S. aureus* challenge	Kim et al. (2010b)
	Murine mAb that targets IsdB	reduces mortality in a murine intravenous challenge model	Brown et al. (2009)
SasX	Immunization with rSasX or α-SasX rabbit serum	reduces size of skin abscesses and severity of acute lung infection; reduces nasal colonization in mice	Liu et al. (2015)
SpA	Vaccine trial with purified SpA_KKAA_, SpA_KKE_ or SpA_KKT_	provides activity against *S. aureus* in murine and guinea pig models of bloodstream infection; reduces *S. aureus* nasal colonization in mice	Kim et al. (2010a), Kim et al. (2015), Sun et al. (2018), Shi et al. (2021)
	Rabbit polyclonal antibody targeting SpA	prevents hyper-inflammatory responses during experimental osteomyelitis	Gehrke et al. (2023)
	Immunization with recombinant or mouse hybridoma-derived SpA_KKAA_-binding mAb	promotes decolonization of mice; therapeutic effect in a renal abscess mouse model; offers protection against neonatal sepsis in mice	Kim et al. (2012), Thammavongsa et al. (2015b), Chen et al. (2019)
	Human mAb specific for SpA	shields mice from *S. aureus* in a bacteremia model	Varshney et al. (2018)
	Humanized α-SpA mAb and Fcγ-engineered antibodies	therapeutic effect against MRSA in a renal abscess mouse model; reduces kidney abscess formation	Chen et al. (2020), Chen et al. (2022)
SraP	Immunization with murine mAb targeting SraP	reduces staphylococcal loads in the bloodstream; improves outcomes of *S. aureus*-mediated sepsis and peritonitis	Vahdani et al. (2021), Zhou et al. (2021)
SrtA	Small molecule inhibitor-based approach (monotherapy with either orientin, punicalagin, rhodionin, scutellarin, or taxifolin)	attenuates staphylococcal virulence during experimental pneumonia	Wang et al. (2021a), Wang et al. (2021c), Song et al. (2022a), Wang et al. (2022a), Wang et al. (2022b)
	Hypodermic injection of chlorogenic acid	reduces mortality of *S. aureus*-infected mice	Wang et al. (2015)
	Infection control by using ML346	protects *Galleria mellonella* larvae from *S. aureus* infection	Guan et al. (2022)
	Acacetin-based therapeutic approach	dampens staphylococcal virulence in a renal abscess mouse model	Bi et al. (2016)
	Anti-infective therapy with either hibifolin, isovitexin, eriodictyol, cyanidin chloride, or chalcone	ameliorates outcomes of staphylococcal lung infection	Zhang et al. (2017a), Wang et al. (2021b), Song et al. (2022b), Su et al. (2022), Tian et al. (2022)
	Therapeutic administration of erianin or 3,6-disubstituted triazolothiadiazole	improves survival of mice following *S. aureus* bloodstream infection	Zhang et al. (2014), Ouyang et al. (2018)
	Administration of an oligopeptide (LPRDA)	protective effect in a mouse model of experimental mastitis	Wang et al. (2018)

a mAb: monoclonal antibody;

b WXD171-IsdB: *Limosilactobacillus reuteri* WXD171 expressing *Staphylococcus aureus* IsdB;

c SpA_KKAA_, SpA_KKA_, or SpA_KKT_: non-toxigenic protein A vaccine variants;

d 3-(4-pyridinyl)-6-(2-sodiumsulfonatephenyl) [1,2,4]triazolo[3,4-b][1,3,4]thiadiazole and related compounds;

e MRSA: methicillin-resistant *Staphylococcus aureus*.

## Concluding remarks

Cell surface proteins are key determinants of *S. aureus* virulence that largely affect host adaptation and immune evasion (Foster et al., 2014; Schneewind and Missiakas, 2019). Undoubtedly, many of these elements modulate host-microbe interaction and essentially contribute to the diverse clinical syndromes *S. aureus* may trigger in mammals (Foster et al., 2014; Schneewind and Missiakas, 2019). Staphylococcal surface proteins may even shape local outbreaks and emergence of new hyper-virulent clones (Li et al., 2012), as well as host tropism as exemplified by the biofilm-associated protein (Bap) which is prevalently expressed in *S. aureus* strains that provoke mastitis in animals (Valle et al., 2020). Notwithstanding, the antigenic variation, diversity, and functional multiplicity of cell surface proteins have hampered attempts to exploit these structures for the development of preventive therapeutics. Although active or passive immunization of laboratory animals conferred protective effects, neutralization of surface proteins may not necessarily represent a suitable approach to prevent staphylococcal infectious diseases in humans. Yet, experimental vaccines and antibody-based immunotherapies that seek to inactivate surface proteins in staphylococci may help to optimize future vaccine trials in diseased individuals. Concomitantly, resolving crystal structures of surface protein-antibody complexes, as recently implemented with ClfA and the mAb tefibazumab (Ganesh et al., 2016), along with an in-depth investigation of non-protective immune imprinting, a phenomenon that correlates with therapeutic failure of IsdB-based immunization trials (Tsai et al., 2022), could assist in the exploitation of *S. aureus* surface proteins for the reformulation of an effective vaccine candidate or fabrication of unique prophylactic tools that foster decolonization of high-risk patients. Ultimately, the discovery of new host ligands of non-excessively studied surface proteins such as the plasmin-sensitive surface protein (Pls), an MRSA-specific cell envelope-displayed glycoprotein (Savolainen et al., 2001; Josefsson et al., 2005; Bleiziffer et al., 2017), may also aid in the design of additional anti-infective strategies and further fuels our knowledge of staphylococcal infection dynamics.

## Author contributions

NS and VW performed the literature review and wrote the manuscript. All authors substantially contributed to the article and approved the submitted version.

## Funding

Work in the VW Laboratory is supported by the German Research Foundation (award WI4582/2-1 to VW; project number 449712894) and the Else Kröner-Fresenius-Stiftung (award 2021_EKEA.16 to VW).

## Conflict of interest

The authors declare that the research was conducted in the absence of any commercial or financial relationships that could be construed as a potential conflict of interest.

## Publisher’s note

All claims expressed in this article are solely those of the authors and do not necessarily represent those of their affiliated organizations, or those of the publisher, the editors and the reviewers. Any product that may be evaluated in this article, or claim that may be made by its manufacturer, is not guaranteed or endorsed by the publisher.
